# High-Pressure Behaviors of Ag_2_S Nanosheets: An in Situ High-Pressure X-Ray Diffraction Research

**DOI:** 10.3390/nano10091640

**Published:** 2020-08-21

**Authors:** Ran Liu, Bo Liu, Quan-Jun Li, Bing-Bing Liu

**Affiliations:** State Key Laboratory of Superhard Materials, Jilin University, Changchun 130012, China; liuran@jlu.edu.cn (R.L.); jluliubo@jlu.edu.cn (B.L.); liquanjun@jlu.edu.cn (Q.-J.L.)

**Keywords:** Ag_2_S, nanosheets, high pressure, phase transitions

## Abstract

An in situ high-pressure X-ray diffraction study was performed on Ag_2_S nanosheets, with an average lateral size of 29 nm and a relatively thin thickness. Based on the experimental data, we demonstrated that under high pressure, the samples experienced two different high-pressure structural phase transitions up to 29.4 GPa: from monoclinic *P*2_1_/*n* structure (phase I, *α*-Ag_2_S) to orthorhombic *P*2_1_2_1_2_1_ structure (phase II) at 8.9 GPa and then to monoclinic *P*2_1_/*n* structure (phase III) at 12.4 GPa. The critical phase transition pressures for phase II and phase III are approximately 2–3 GPa higher than that of 30 nm Ag_2_S nanoparticles and bulk materials. Additionally, phase III was stable up to the highest pressure of 29.4 GPa. Bulk moduli of Ag_2_S nanosheets were obtained as 73(6) GPa for phase I and 141(4) GPa for phase III, which indicate that the samples are more difficult to compress than their bulk counterparts and some other reported Ag_2_S nanoparticles. Further analysis suggested that the nanosize effect arising from the smaller thickness of Ag_2_S nanosheets restricts the relative position slip of the interlayer atoms during the compression, which leads to the enhancing of phase stabilities and the elevating of bulk moduli.

## 1. Introduction

As a well-known metal sulfide, Ag_2_S is a direct narrow band gap semiconductor (~1.5 eV), with high absorption coefficient (10^4^ m^−1^), good chemical stability and optical properties [1,2,3]. So far, Ag_2_S materials have been extensively synthesized and studied due to their important applications including semiconductors, photovoltaic cells, infrared detectors, photoelectric switches and oxygen sensors [4,5,6]. Recently, the phase transitions of Ag_2_S have attracted much attention and a lot of research has been conducted around this topic. Under ambient conditions, Ag_2_S is stable in a monoclinic structure with *P*2_1_/*n* space group (α-Ag_2_S) [7]. While heating above 450 K, Ag_2_S undergoes a thermo-induced phase transition and reforms into a body-centered cubic (bcc) structure (β-Ag_2_S, space group *Im*$\bar{3}$*m*) [8,9]. In this high-temperature structure, silver ions are randomly distributed over the interstitial sites of a bcc sulfur lattice [10,11], leading to a favorable ionic conductivity as high as 5 Ω^−1^ cm^−1^ [12]. Thereby, β-Ag_2_S is considered as a fast ionic conductor (FIC), which has potential applications including in energy, analytical chemistry, biomedicine, solid-state ionic devices and so forth [13,14,15]. At about 860 K, Ag_2_S further converse into a face-centered cubic (fcc) phase (γ-Ag_2_S), then keeping stable up to melting temperature [9].

Instead of temperature, high pressure is an effective approach for tuning both structural phase transitions and physical properties [16,17]. Under high pressure, the atomic arrangement of materials would be dramatically changed, and thus, the electronic structures and physical properties could be improved. For bulk Ag_2_S, structural and optical behaviors under high pressure have been systematically studied by synchrotron X-ray diffraction and infrared spectroscopy measurements [15,18,19]. It is demonstrated that Ag_2_S bulk material experiences a series of phase transitions up to 40 GPa. At 5.1 GPa, bulk Ag_2_S transforms from the *P*2_1_/*n* structure (α-Ag_2_S, phase I) to an orthorhombic *P*2_1_2_1_2_1_ structure (phase II). Elevating the pressure up to 8.8 GPa, Ag_2_S undergoes the second phase transition, from phase II to a monoclinic *P*2_1_/*n* structure (phase III), which is isosymmetric to phase I. At 28.4 GPa, phase III further transforms into a *Pnma* structure (phase IV). Accompanied by structural transformations, the band gap of Ag_2_S is narrowed gradually by increasing the pressure. Beyond 22 GPa, the pressure effectively tunes the semiconducting Ag_2_S into a metal [19].

Up to now, with the rapid development of synthesis technology, many kinds of nanostructured Ag_2_S have been prepared, such as quantum dots [20], nanoparticles [21,22], nanotubes [23], nanowires [24], nanorods [25], sheet-like and cube-like nanocrystals [26] and so forth. This progress inspired researchers to begin to focus on the pressure-induced phase transitions of Ag_2_S nanomaterials, where they expected to obtain novel properties under pressure. Thus far, there has been one report on the high-pressure behaviors of pure and Y-doped Ag_2_S nanoparticles [27]. The particle sizes of the two samples were estimated to be about 30 nm. Indeed, Wang et al. have found that both of the samples exhibit slightly higher transition pressure values and obviously increased bulk moduli than that of the corresponding bulk materials. We note that previous studies have been focused on Ag_2_S nanocrystals with random geometric shapes. It is still unclear what behaviors of Ag_2_S nanocrystals with special morphologies will actually be induced by high pressure. In particular, it is still unknown whether or not they will choose the known structural evolution laws as well as the nanoparticles. These require further studies.

In this work, we investigate the high-pressure behaviors of Ag_2_S nanomaterials with sheet-like morphologies using the in situ high-pressure X-ray diffraction up to about 30 GPa. A set of phase transitions of Ag_2_S nanosheets were observed. Interestingly, the Ag_2_S nanosheets exhibit even higher structural stability and lower compressibility under high pressure, remarkably different from the Ag_2_S bulk materials and nanoparticles. Further analysis suggested that the stronger nanosize effect arising from the smaller thickness of Ag_2_S nanosheets effectively restricts the relative position slip of the interlayer atoms during the compression, which leads to the enhancement of phase stabilities and the elevation of bulk moduli. Our findings give a further insight into high-pressure behaviors of Ag_2_S nanomaterials.

## 2. Materials and Methods

In this study, Ag_2_S nanosheet was provided by Tongshun Wu, Ph. D. of State Key Laboratory of Inorganic Synthesis and Preparative Chemistry, Jilin University (Changchun, China), which was prepared by solvothermal method. The initial sample was dispersed in ethanol, with no substrate or coating. A HITACHI H-8100 (Hitachi, Ltd., Tokyo, Japan) transmission electron microscope (TEM) with accelerating voltage of 200 kV was employed to analyze the typical product. X-ray powder diffraction (XRD) was used to characterize the product and the XRD patterns at ambient condition were collected by a Rigaku D/max-rA XRD (Rigaku Co., Tokyo, Japan) spectrometer (Cu, *Kα*, *λ* = 1.5406 Å).

A symmetric diamond anvil cell (DAC) with flat diamond culets of 400 μm in diameter was used to generate high pressure. The DAC allowed access to the X-ray diffraction angular range 4*θ* = 50°. A sheet of T301 stainless steel was selected as the gasket and pre-indented in the DAC to an initial thickness of about 40 μm. Then, a hole of 150 μm in diameter was drilled manually by a tungsten carbide needle in the middle of the dent, which served as a sample chamber. The Ag_2_S nanosheet samples along with the dispersant were loaded simultaneously into the sample chamber to keep the dispersity. A mixture of methanol and ethanol at a ratio of 4:1 was used as the pressure transmitting medium. A tiny ruby ball ~5 μm in diameter was loaded in the sample chamber, and pressure was determined by the frequency shift of the ruby *R_1_* fluorescence line. High-pressure X-ray diffraction experiments were carried out up to 30 GPa using a synchrotron X-ray source (λ = 0.386 Å) of the beamline X17C of National Synchrotron Light Source (NSLS), Brookhaven National Lab (BNL). The monochromatic X-ray beam was focused down to 25 μm × 25 μm using Kirkpatrick-Baez mirrors. For filtering out the tail of the X-ray beam, a pinhole was placed before the sample position as a cleanup aperture. The X-ray diffraction images were collected by a MAR345 image plate, located ~350 mm behind the sample. Exposure times were typically 300~600 s. Geometric correction and radial integration was done using the Fit2D software (v12.077, GRENOBLE, France). The observed intensities were integrated as a function of 2*θ* in order to give conventional, one-dimensional diffraction profiles. Rietveld refinement was performed by GSAS-EXPGUI package (v1.80, Gaithersburg, Maryland, USA). A Birch-Murnaghan (BM) equation of state (EOS) was employed to fit the experimental *P-V* relationship, using Origin Pro software (v8.0, Northampton, MA, USA).

## 3. Results and Discussion

**Figure 1:**
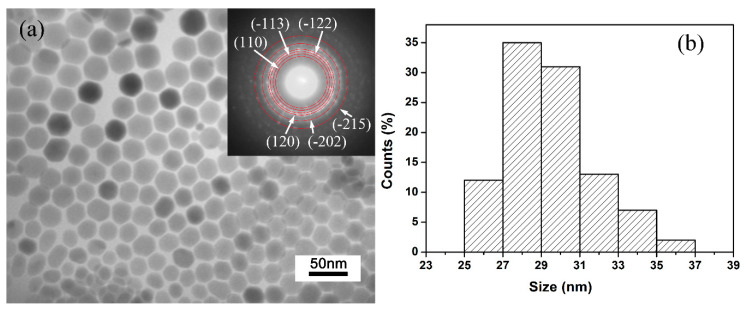
Typical transmission electron microscope (TEM) images and particle size distribution: (**a**) TEM images and selected area electron diffraction (SAED) image (inset) of the samples. (**b**) Size distribution of Ag_2_S nanosheets.

To investigate the morphology and structure of the Ag_2_S samples, TEM and selected area electron diffraction (SAED) analysis was performed. Figure 1a shows the TEM image and SAED pattern of the Ag_2_S samples. It can be clearly seen that all Ag_2_S nanocrystals are well dispersed and possess sheet-like morphology; no other shapes can be observed. SAED pattern (inset in Figure 1a) determines the samples are well crystalized in a monoclinic *P*2_1_/*n* structure. Figure 1b shows the lateral size distribution of Ag_2_S nanosheets. The distribution is relatively narrow, and the average lateral size is 29 ± 2 nm. In principle, for the TEM image, the contrast of the image normally represents the thickness of samples. The smooth contrast of each Ag_2_S nanosheet grain proves that the samples have uniform thickness. Interestingly, the Ag_2_S nanosheets are approximately semitransparent (see the sample overlaps in TEM image), which demonstrates that the thickness of samples is relatively thin, far below 29 nm. All the analyses show that Ag_2_S samples are well crystallized and have an almost-uniform morphology as nanosheets, providing ideal samples for high-pressure research.

**Figure 2:**
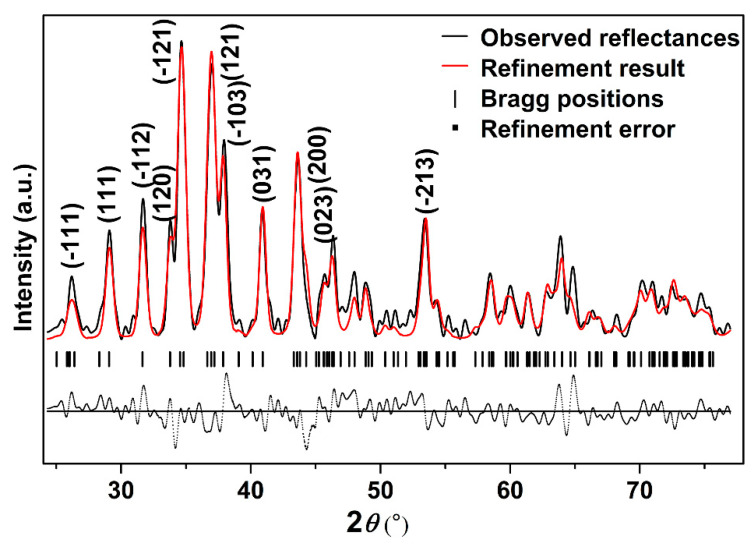
The typical X-ray powder diffraction (XRD) pattern of Ag_2_S nanosheets under ambient conditions; *Rwp* = 0.3133, *Rp* = 0.2028, and *chi^2* = 84.89.

In order to further study the crystal structure of Ag_2_S nanosheets, XRD analysis was carried out. Figure 2 shows the typical XRD pattern of the samples and its Rietveld refinement under ambient conditions. It can be seen that the diffraction pattern can be indexed into α-Ag_2_S phase (Joint Committee on Powder Diffraction Standards (JCPDS) card no. 14-0072) (monoclinic structure, space group *P*2_1_/*n*). Vertical markers indicate the Bragg reflections of the *P*2_1_/*n* monoclinic structure. It can be seen that no other diffraction peaks exist, which means that no other phases or other impurities are mixing in the sample. The Rietveld refinement of the XRD pattern at ambient pressure yields *a* = 4.214 ± 0.001 Å, *b* = 6.912 ± 0.001 Å, *c* = 7.853 ± 0.001 Å, *β* = 99.580° ± 0.010°, *V_0_* = 225.55 ± 0.034 Å^3^ for Ag_2_S nanosheets, slightly smaller than 30 nm Ag_2_S nanoparticles and bulk materials [27,28]. The existence of a lattice shrinking phenomenon of Ag_2_S nanosheets could be attributed to the stronger nanosize effect as a result of their smaller thickness.

**Figure 3:**
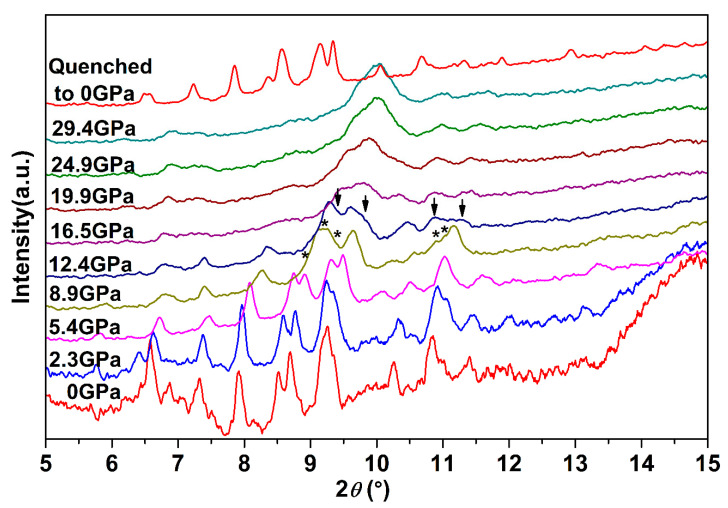
Synchrotron X-ray diffraction patterns of Ag_2_S nanosheets under different pressures. The asterisks and arrows denote the occurrences of new peaks for phase II and phase III, respectively.

**Figure 4:**
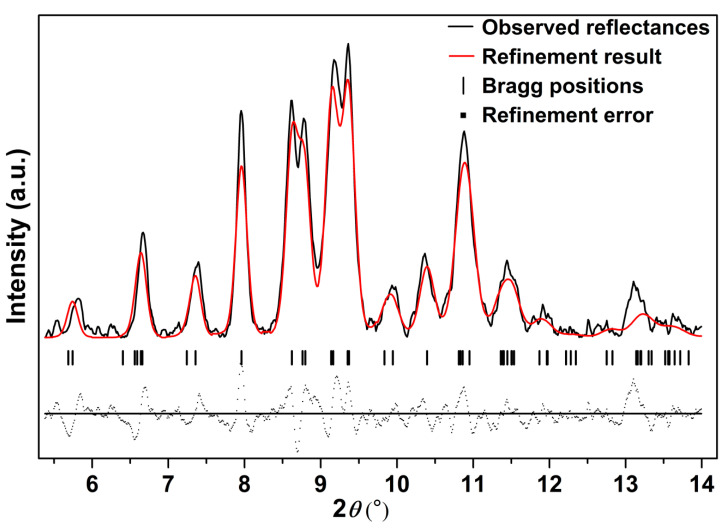
The refinement result for the monoclinic *P*2_1_/*n* structure (phase I) of Ag_2_S nanosheets at 5.38 GPa, *Rwp* = 0.3011, *Rp* = 0.1836 and *chi^2* = 109.9.

To systematically explore the high-pressure behavior of Ag_2_S nanosheets, we carried out an in situ high-pressure X-ray diffraction study. Figure 3 shows synchrotron X-ray diffraction patterns of Ag_2_S nanosheets under different pressures. It is clear that under high pressures, two phase transitions for the samples are observed up to 29.4 GPa. With pressure increasing, all the peaks of *α*-Ag_2_S phase shift to smaller *d*-spacing, indicating a pressure-induced reduction of *d*-spacing and shrinkage of the unit cell. The initial phase can be maintained up to 8.9 GPa, much more stable than that in Ag_2_S nanoparticles and bulk materials [19,27]. At 5.4 GPa (the first phase transition point of bulk Ag_2_S), the measured XRD pattern is consistent with the starting monoclinic *P*2_1_/*n* structure (phase I). The mutual agreements encourage us to perform subsequent fine Rietveld analysis on our experimental data by using the *P*2_1_/*n* structure. As shown in Figure 4, the fitting gives a successful result to the experimental data. The lattice constants of Ag_2_S nanosheets at 5.38 GPa are as follows: *a* = 4.107 ± 0.002 Å, *b* = 6.659 ± 0.003 Å, *c* = 7.616 ± 0.004 Å, *β* = 99.62° ± 0.03, *V_0_* = 205.3 ± 0.1 Å^3^. According to the fitting data, all of the diffraction peaks can be indexed to the initial monoclinic *P*2_1_/*n* structure. No new phases were observed at this pressure.

While compressing up to 8.9 GPa, several new peaks (marked as asterisks) start to emerge obviously and their intensity grows with pressure increasing (see Figure 3), indicating that *α*-Ag_2_S undergoes a phase transition. According to previous reports, these new peaks located at 8.96°, 9.18°, 9.39°, 10.90° and 11.05° can be indexed into the (120), (112), (121), (113) and (201) diffractions of orthorhombic *P*2_1_2_1_2_1_ structure (phase II). Both phase I and phase II of Ag_2_S coexist under this pressure. No pure phase II structure could be obtained in our work. With continuous compression, all the peaks of ambient phase I became weak gradually.

**Figure 5:**
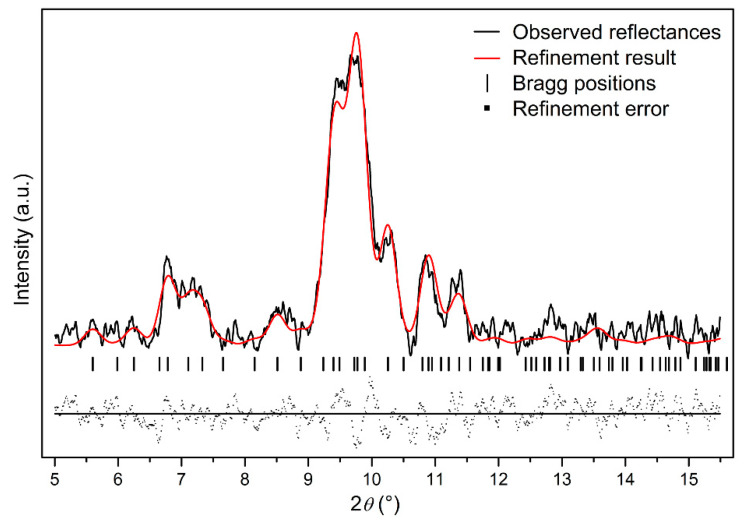
The refinement result for phase III of Ag_2_S nanosheets at 16.5 GPa, *Rwp* = 0.1921, *Rp* = 0.1402 and *chi^2* = 67.78.

Further compressed to 12.4 GPa, it can be clearly seen that a set of new diffraction peaks (2*θ* = 9.39°, 9.78°, 10.87°, 11.30°) emerged (marked as arrows in Figure 3), indicating that Ag_2_S nanosheets undergo the second phase transition. These new diffraction peaks can be indexed into (022), (12-1), (200) and (014) diffractions of monoclinic *P*2_1_/*n* structure (phase III), isosymmetric to the ambient phase. Both phase II and phase III coexist under this pressure. However, the peaks corresponding to phase II lost a lot of intensity and then completely disappeared at the pressure of 16.5 GPa. Under this pressure, all of the remaining peaks can be indexed to phase III, indicating Ag_2_S nanosheets crystallized in monoclinic *P*2_1_/*n* structure completely. Typical Rietveld refinement for phase III is plotted in Figure 5, yielding *a* = 4.079 ± 0.003 Å, *b* = 5.800 ± 0.006 Å, *c* = 7.948 ± 0.008 Å and *β* = 93.15° ± 0.07 (*V* = 187.7 ± 0.2 Å^3^). Compressing the samples up to 29.4 GPa, the highest pressure in our study, no new diffraction peaks were observed, Ag_2_S nanosheets are mainly still maintaining in phase III.

In previous studies, we noted that bulk Ag_2_S undergoes the phase transition sequence *P*2_1_/*n* (phase I) → *P*2_1_2_1_2_1_ (phase II) → *P*2_1_/*n* (phase III) → *Pnma* (phase IV) at pressures of 5.1GPa, 8.8 GPa and 28.4 GPa, respectively [19]. For pure 30 nm Ag_2_S nanoparticles, the transition pressures elevate slightly compared to the bulk: from phase I to phase II at about 6.83 GPa and then to phase III at 9.3 GPa [27]. Compared to these reports, we noted that phase transition pressure in Ag_2_S nanosheets was about 2–3 GPa higher than that in 30 nm Ag_2_S nanoparticles and bulk crystals. Moreover, phase IV [19], which formed at 28.4 GPa in bulk counterparts, was not observed in our samples. Thus, our work reveals that Ag_2_S nanosheets exhibit a different high-pressure property compared with previous reports. According to the analysis in the TEM image before, it was shown that the Ag_2_S nanosheets have a relatively thin thickness, far below 29 nm (the average size of the sample). Therefore, we suggest that the stronger nanosize effect arising from the smaller thickness of Ag_2_S nanosheets effectively restricts the relative position slip of the interlayer atoms during compression, which leads to the enhancing of phase stabilities.

**Figure 6:**
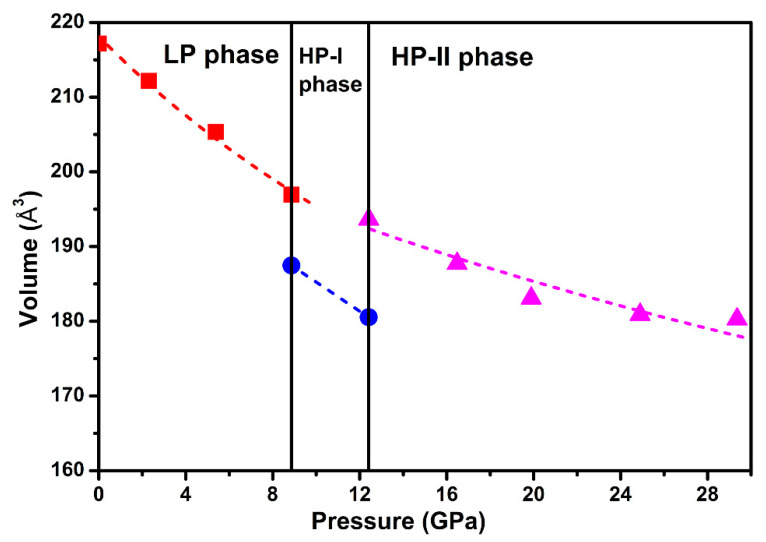
Room-temperature equation of state data for Ag_2_S nanosheets. A third-order Birch-Murnaghan (BM) equation of state (EOS) is introduced to fit our pressure-volume data.

Figure 6 shows the compressive behavior of Ag_2_S nanosheets. A third-order BM EOS was introduced to fit our pressure-volume data. For monoclinic *P*2_1_/*n* structure (phase I), by fixing the first pressure derivative of isothermal bulk modulus (*B*_0_*′*) to 4, the bulk modulus (*B*_0_) was obtained as 73(6) GPa. The obtained bulk modulus *B_0_* for phase I, the ambient phase, was much higher than that of the bulk Ag_2_S (*~*34–38(2) GPa), even higher than that of the 30 nm Ag_2_S nanoparticles (*B*_0_ = 57.3(6) GPa) [19,27]. For phase III, during the fitness, the values of the bulk modulus (*B*_0_) and zero pressure cell volume (*V*_0_) were left to vary freely, and the first derivative of *B*_0_ with pressure (*B*_0_*′*) was fixed to 4. As shown in Figure 6, the fitting to the *P-V* curve yields a bulk modulus *B*_0_ of 141(4) GPa and unit cell volume *V*_0_ of 207(6) Å^3^. Compared to previous studies, the obtained bulk modulus *B*_0_ for phase III of Ag_2_S nanosheets was also larger than the bulk counterparts (102(4) GPa) and 30 nm nanoparticles (123.1(0) GPa) [19,27]. According to the above analysis, we consider that Ag_2_S nanosheets exhibit significant difference in compression properties under high pressure, a much lower volume compressibility than previous reports. A similar phenomenon was observed in some other nanomaterials, e.g., C_60_ nanosheets and ZnO nanocrystal [29,30], and has potential benefits in keeping the stability of materials. It is well known that nanosize effect can strongly influence the bulk modulus; thereby, the 30 nm nanoparticles exhibit higher bulk modulus than the bulk materials. In our experiment, the Ag_2_S nanosheets have a very small size in thickness, much lower than their grain size of 29 nm. Thus, the stronger nanosize effect from our samples may cause the bulk modulus to largely elevate.

**Figure 7:**
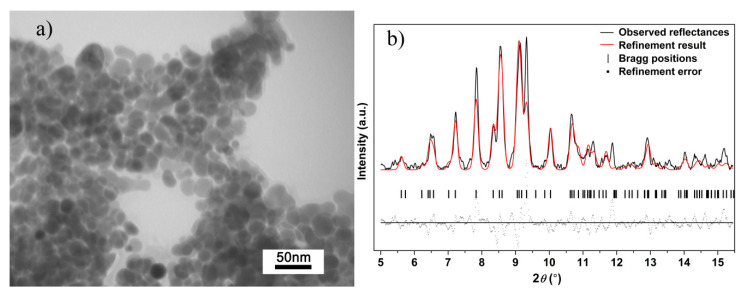
(**a**) TEM images and (**b**) Rietveld refinement result of Ag_2_S nanosheets quenched to ambient conditions; *Rwp* = 0.3982, *Rp* = 0.2929 and *chi^2* = 160.3.

In our high-pressure research, the quench process is implemented. TEM images and XRD pattern of Ag_2_S nanosheets quenched to the ambient conditions are shown in Figure 7. According to the TEM image of the quenched sample, although Ag_2_S samples could not maintain their uniform shape because of the high pressure and high shear stresses, it still keeps the sheet-like morphology in nanoscale. Based on this result, we can reasonably draw a conclusion that during the compressing process, Ag_2_S samples in our experiment remain nanomaterials, and no agglomeration took place. After decompressing to ambient pressure, the shapes of all the diffraction peaks for Ag_2_S nanosheets return to the initial monoclinic *P*2_1_/*n* structure (phase I), indicating the reversibility of the pressure-induced structural transitions. Further analysis showed that the (-112), (-103) peak was enhanced after compression. The effect of non-hydrostatic pressure and high pressure gives nanosheet samples a certain orientation and texture.

## 4. Conclusions

In summary, we studied the high-pressure behaviors of Ag_2_S nanomaterials with sheet-like morphologies using in situ high-pressure X-ray diffraction up to about 30 GPa. Under high pressure, Ag_2_S nanosheets undergo the phase transition sequence *P*2_1_/*n* (phase I) → *P*2_1_2_1_2_1_ (phase II) → *P*2_1_/*n* (phase III) at pressures of 8.9 GPa and 12.4 GPa. Interestingly, the Ag_2_S nanosheets exhibit a wide field of structural stability and much lower compressibility under high pressure, remarkably different from the corresponding bulk materials and 30 nm nanoparticles. Further analysis suggests that a stronger nanosize effect arising from smaller thickness in Ag_2_S nanosheets effectively restricts the relative position slip of the interlayer atoms during the compression, which leads to the enhancing of phase stabilities and the elevating of bulk moduli. Our findings give a further insight into high-pressure behaviors in Ag_2_S nanomaterials.
